# Understanding lactate and its clearance during extracorporeal membrane oxygenation for supporting refractory cardiogenic shock patients

**DOI:** 10.1186/s12872-023-03221-y

**Published:** 2023-06-16

**Authors:** ER Kurniawati, PW Weerwind, JG Maessen

**Affiliations:** 1 Department of Cardiothoracic Surgery, Maastricht University Medical Center+, PO Box 5800, P. Debyelaan 25, 6202 AZ Maastricht, the Netherlands; 2 Cardiovascular Research Institute Maastricht (CARIM), Maastricht University, Maastricht, the Netherlands

The recent study by Scolari et al. [1] entitled ‘Association between serum lactate levels and mortality in patients with cardiogenic shock receiving mechanical circulatory support: a multicenter retrospective cohort study’, published in the *BMC Cardiovascular Disorders* evaluates the prognostic role of serum lactate and lactate clearance over time in cardiogenic shock patients treated with mechanical circulatory support. The authors concluded that serum lactate levels are an important prognostic biomarker for 30-day mortality in cardiogenic shock patients treated with temporary mechanical circulatory support, i.e., the Impella CP device or venoarterial extracorporeal membrane oxygenation (ECMO). However, this study did not consider the impact of the two different types of mechanical circulatory support on the lactate kinetics in these critically ill patients. Consequently, the contextual interpretation of the findings in a complex and dynamic concept of lactate clearance during cardiogenic shock remains unclear.

Serum lactate and its clearance have been proven to be reliable independent markers of illness severity and mortality in critically ill patients [2, 3]. However, it should be noted that other factors unrelated to tissue oxygenation (e.g., seizures, diabetic ketoacidosis, burns and smoke inhalation, liver dysfunction, genetic, drugs administration, etc.) might elevate lactate levels [4, 5]. Lactate levels are the result of a shift to an anaerobic metabolism pathway and its clearance through the liver and kidneys. To have an adequate lactate clearance, sufficient oxygen delivery is indicated for tissue perfusion recovery and oxygen debt repayment. The latter can be defined as the extra oxygen that must be used in the oxidative energy process after a period of hypoxia to reconvert lactic acid to glucose and decomposed adenosine triphosphate as well as creatine phosphate to their original states. Scolari et al. [1] did not indicate if all patients, in both the survivor and non-survivor groups, equally received sufficient oxygen delivery. Besides, the Impella CP device only provides circulatory support by unloading the left ventricle, whereas venoarterial ECMO provides both circulatory and respiratory support. Nonetheless, Scolari et al. [1] reported an obvious improvement in lactate level after initiation of mechanical circulatory support, reflecting a hemodynamic response. They rightfully suggested that this improvement in lactate might have greater prognostic utility than initial lactate levels.

Although the end result is important, understanding the process is conjointly important to achieve more favorable outcomes. For example, patients with higher initial lactate levels should receive a higher oxygen delivery compared to patients with lower initial lactate levels in order to repay the oxygen debt. To do so, cardio-respiratory support using ECMO should be initiated timely by using a larger cannula. Often, this is unattainable due to vascular restrictions [6]. Hence, further studies focusing on the dynamics of oxygen debt repayment rather than solely lactate levels and its clearance will be valuable to understand this complex topic during temporary mechanical circulatory support.

We are grateful to Scolari and colleagues [1] for sharing their experience and knowledge in this commendable multicenter retrospective cohort study and for giving an important insight into such a complex and dynamic concept of lactate clearance in a cardiogenic shock setting.

## Data Availability

Not applicable as no datasets were generated or analyzed during the current study.

## Abbreviation

ECMO: Extracorporeal Membrane Oxygenation
